# A novel characterisation approach to reveal the mechano–chemical effects of oxidation and dynamic distension on polypropylene surgical mesh†

**DOI:** 10.1039/d1ra05944k

**Published:** 2021-10-27

**Authors:** Nicholas T. H. Farr, Sabiniano Roman, Jan Schäfer, Antje Quade, Daniel Lester, Vanessa Hearnden, Sheila MacNeil, Cornelia Rodenburg

**Affiliations:** Department of Materials Science and Engineering, University of Sheffield Sir Robert Hadfield Building, Mappin Street UK n.t.farr@sheffield.ac.uk; Insigneo Institute for in silico Medicine The Pam Liversidge Building, Sir Robert Hadfield Building, Mappin Street Sheffield UK; Leibniz Institute for Plasma Science and Technology (INP e.V.) Felix-Hausdorff-Str. 2 17489 Greifswald Germany; Polymer Characterisation Research Technology Platform, University of Warwick Library Road CV4 7AL Coventry UK

## Abstract

Polypropylene (PP) surgical mesh, used successfully for the surgical repair of abdominal hernias, is associated with serious clinical complications when used in the pelvic floor for repair of stress urinary incontinence or support of pelvic organ prolapse. While manufacturers claim that the material is inert and non-degradable, there is a growing body of evidence that asserts PP fibres are subject to oxidative damage and indeed explanted material from patients suffering with clinical complications has shown some evidence of fibre cracking and oxidation. It has been proposed that a pathological cellular response to the surgical mesh contributes to the medical complications; however, the mechanisms that trigger the specific host response against the material are not well understood. Specifically, this study was constructed to investigate the mechano–chemical effects of oxidation and dynamic distension on polypropylene surgical mesh. To do this we used a novel advanced spectroscopical characterisation technique, secondary electron hyperspectral imaging (SEHI), which is based on the collection of secondary electron emission spectra in a scanning electron microscope (SEM) to reveal mechanical–chemical reactions within PP meshes.

## Abstract 2

The application of novel SEM-based spectroscopical characterisation techniques reveals the mechano–chemical effects of oxidation and dynamic distension on polypropylene surgical mesh.

## Graphical abstract

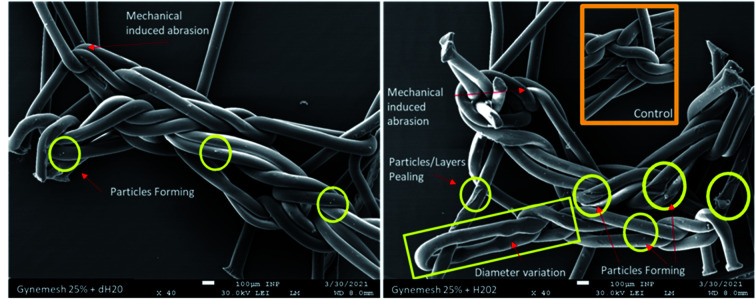

## 1. Introduction

Implantable mesh made of polypropylene (PP) fibres has been used surgically to treat soft tissue repair since the 1950s. This surgical mesh designed with a knitted pattern has been used to treat complex hernias and other abdominal wall procedures. Then in the 1970s, the same mesh was introduced for pelvic floor repair.^1^ Several corporations around the globe designed and manufactured a wide range of different mesh products for these medical applications representing a huge market in the medical device field.^1^ Unfortunately, the mesh was not tested for the site of implantation on the assumption that a mesh that worked well in the abdomen would also work well in the pelvic floor. This assumption has proved to be incorrect and patients have suffered from sustained inflammation around the mesh, with pain, mesh contraction and even extrusion of mesh through patient's tissues.^1^ Regulators have now banned the use of the PP mesh for most pelvic procedure in countries including New Zealand, Australia and the UK, and the safety of these products has been questioned by health agencies in the US and Canada. Prior to this recent ban manufacturers developments focused on the method used to introduce these devices and more recently new designs were produced to reduce the mesh density and the knitted pattern was modified to achieve a more flexible material resulting in better compliance with the patients tissues.^2^ However, the material used to produce these meshes is the same type of PP fibres used 60 years ago based on the long-term assumption that it is an inert material.

After prolapse surgery, extrusion of the mesh through the vaginal wall occurs in at least 10% of patients and can develop years after mesh insertion.^3^ Two principal theories are proposed to explain these complications: (i) a mechanical mismatch between the properties of the native tissues and the mesh which does not allow elastic deformation after changes in intra-abdominal pressures; and (ii) an adverse cellular response to the fibres of the PP mesh itself.^4^ These theories are not mutually exclusive. In explanted material, an adverse foreign body response to the mesh has been observed, characterised by macrophage activation promoting scar like tissue formation.^5^ This fibrotic response causes the encapsulation and contraction of the mesh, with pain and extrusion through the vaginal tissues in the worst cases.^6^ However, the exact mechanisms that trigger the specific host response are not well understood. It is likely to be multi-factorial where PP fibres, their mesh pattern and inexpert surgical implantation into a challenging wound bed (the post-menopausal vagina) can all contribute to a medical device producing an unacceptably high rate of clinical complications.^7^

There is growing evidence that the nature of the polymer material selected may not be suited for this purpose. Despite manufacturers presentation of the PP mesh materials as inert materials, surface oxidation of these fibres has been identified using analytical techniques such as Fourier transform infrared spectroscopy (FTIR) and X-ray photoelectron spectroscopy (XPS) in meshes explanted from patients with clinical complications.^8,9^ These studies provide evidence which questions whether the PP material is inert in the implanted environment. The mechanism by which PP meshes fail post implantation is still open to debate, evidence has been presented that points at environmental oxidation degradation (*i.e.* stress-corrosion) as a potential option. Kurtz *et al.*, showed that the secretion of reactive oxygen species (ROS) from immune cells attached to the PP fibres may lead to surface oxidation and, consequently, embrittlement of the PP material.^10^ Imaging techniques, such as scanning electron microscope (SEM), have shown surface degradation of PP and transverse cracking on the surface of PP.^8,9,11–14^ However, this is a finding contested by the manufacturers who have claimed all such reports are based on contamination by fixation artefacts.^15^

To exclude any possibility of contamination artefacts this study used the novel and advanced spectroscopic characterisation technique secondary electron hyperspectral imaging (SEHI) which is based on the collection of secondary electrons emission spectra in a SEM^16^ at low primary beam energies, and allows the chemical inspection of uncoated polymer surfaces at multiple length scales. This unique approach permitted key insights into changes in chemical bonding taking place in PP meshes on the nanoscale *via* mechano–chemical processes by building high resolution chemical maps. The SEHI technique has been successfully applied to a range of organic and inorganic material systems^17–19^ however this is the first study to apply SEHI to pelvic floor materials. Applications of SEHI include the analysis of molecular orientation within organic electronic devices, semi-crystalline polymers for functional group chemical mapping and revealing variations in nanostructures that form natural materials.^17,19–22^ The advantage of SEHI over traditional surface analysis methods such as XPS is that local variations in chemical alterations occurring at the nanoscale can be observed, variations which can be hidden in large surface area analysis techniques. Local variations in chemical structures and changes to surface topography are expected to significantly affect cell material interactions of pelvic floor materials following implantation in the body, including the response of macrophages and initiation of a foreign body response.

A previous paper from our laboratory^23^ demonstrated that dynamic distention of PP mesh materials for just 3 days causes irreversible distortion and failure in the mechanical properties of these PP bulk materials, however, the underlying mechanism remained unclear and did not account for the oxidative environment. Previous studies have looked at the effect of distention^24,25^ or oxidative stress *in vitro*,^9^ this is the first study that combines both to reproduce the conditions expected to occur *in vivo*. The aim of this study was to evaluate two commercial PP products (Gynemesh® and Restorelle®) classified as high density and low density mesh respectively. Materials were subjected to mechanical distention using a dynamic bioreactor combined with oxidative stress (achieved by placing samples in a solution of hydrogen peroxide (H_2_O_2_)) following a slight modification of the accelerated degradation test required for regulatory approval of these devices.^26^ After testing, SEHI was carried out on fibre surfaces and cross-sections of mesh in multiple areas in order to assess changes to the materials resulting from combined mechanical and oxidative stress exposure.

## 2. Materials and methods

### 2.1 Materials

Two commercially available materials fabricated from PP were used for comparison, a heavy weight PP mesh, Gynemesh® (Johnson & Johnson) and an ultra-light weight PP mesh, Restorelle® (Coloplast, Humlebaek, Denmark).

### 2.2 Sample preparation

Strips of material were cut using sterilised scissors within a cell culture cabinet along the longitudinal direction of the surgical mesh. Due to the differences in the knitted design of the two products, Gynemesh was cut to 14 mm × 9 mm, and Restorelle was cut to 14 mm × 6 mm.

### 2.3 Oxidative stress induced by an accelerated degradation test

This study was conducted based on ISO 10993-13, Biological Evaluation of Medical Devices – Part 13: Identification and Quantification of Degradation Products from Polymeric Medical Devices.^26^ Samples were placed in triplicates within a 6 well plate and covered with the appropriate volume (test article : solution ratio of 1 g : 10 mL) of 3% hydrogen peroxide (hydrogen peroxide solution, contains inhibitor, 30 wt% in H_2_O, ACS reagent, Sigma-Aldrich). In a change from the standard test, samples were incubated at 60 °C for 14 days without agitation (testing conditions included in Table 1).

**Table 1:** Conditions used in experiments conducted under dynamic distention, under an oxidative stress or under combination of both. Experiments were run in triplicates (3 samples of the same material in each chamber of the bioreactor or well plate) and each condition was run twice for both materials (one triplicate for tensile test and the other triplicate for the rest of the analyses)

Conditions for both materials				
Solution	Time	Distention	Temperature	Sample ID
dH_2_O	6 hours	No distention	37 °C	Control
dH_2_O	6 hours	5%	37 °C	5% 6 hours in dH_2_O
dH_2_O	6 hours	25%	37 °C	25% 6 hours in dH_2_O
dH_2_O	6 hours	50%	37 °C	50% 6 hours in dH_2_O
dH_2_O	72 hours	25%	37 °C	dH_2_O at 25% 3 days
H_2_O_2_	72 hours	25%	37 °C	H_2_O_2_ at 25% 3 days
dH_2_O	14 days	No distention	60 °C	dH_2_O 14 days
H_2_O_2_	14 days	No distention	60 °C	H_2_O_2_ 14 days

### 2.4 Dynamic distention with bioreactor

**Fig. 1:**
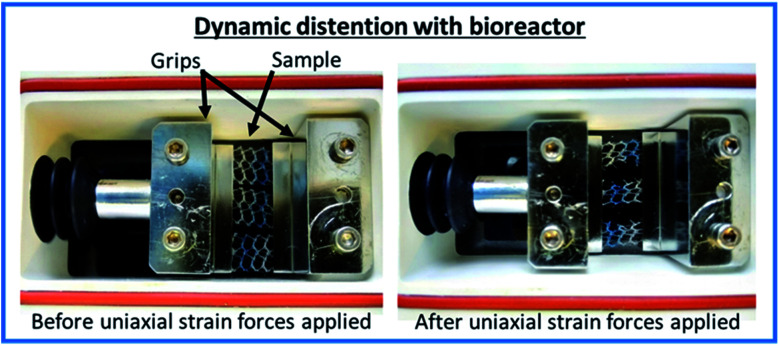
Annotated images of the experimental set up of the TC-3 load bioreactor (Ebers Medical Technology SL, Zaragoza, Spain).

Samples were clamped flat with a distance of 8 mm between the two grips of a TC-3 load bioreactor (Ebers Medical Technology SL, Zaragoza, Spain) along the longitudinal direction of the surgical mesh (see Fig. 1). Samples were then submerged in 25 mL solution and subjected to cyclic uniaxial distension with a frequency of 0.5 cycles per minute (to mimic the number of times intra-abdominal pressures are increased during normal breathing) at 37 °C following the conditions described in Table 1, and as described in reference.^27^

### 2.5 Uniaxial tensile testing

**Fig. 2:**
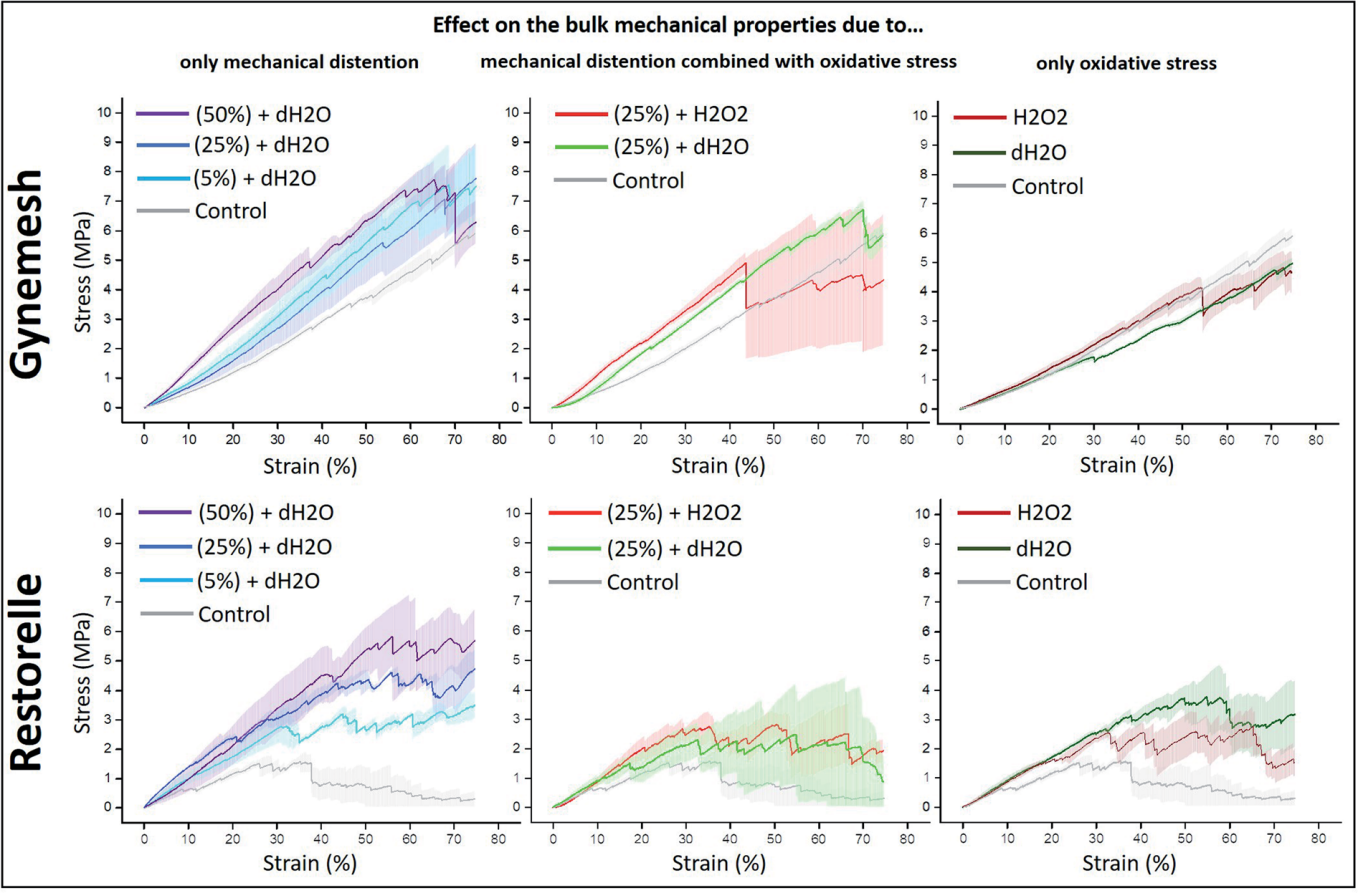
Stress–strain curves of Gynemesh (top) and Restorelle (bottom) after; (left) 5%, 25% and 50% dynamic distention for 6 hours; (middle) 25% dynamic distention in dH_2_O or H_2_O_2_ for 3 days; and (right) 14 days incubation at 60 °C within dH_2_O or H_2_O_2_; all in comparison to that of the control material.

Uniaxial ramp testing was performed for all samples after accelerated degradation and dynamic distention conditions. A tensiometer (BOSE Electroforce test instruments, Minnesota, USA) was used. Samples (*n* = 3, for each material and group) were clamped between two grips of the tensiometer with a testing length of 8 mm. A load cell of 450 N was used. The commercial meshes were loaded in the longitudinal direction, in the direction of use as indicated by the manufacturer. A tensile test was then applied at a rate of 0.1 mm s^−1^ and a displacement of 7 mm. The values for load and displacement were displayed as stress (*y* axis, MPa) *vs.* strain (*x* axis, % of displacement) plots (Fig. 2) after being normalised by area (width *x* thickness) and the length of the sample respectively. All experiments were performed under constant laboratory conditions (23 °C, British air humidity 80%).

### 2.6 Conventional SEM imaging

Observation of the surface morphology of the Gynemesh and Restorelle was performed using a scanning electron microscope (FEI Nova Nano 450 SEM). Samples were not subject to deposition of conductive coatings, in contrast to usual polymers SEM analysis practice. To avoid surface charging and consequent damage to the sample a low accelerating voltage (1 kV) with typical vacuum pressure of 10^−5^ mbar at a working distance of 3 mm was applied. An Everhart–Thornley detector (ETD) for low magnification images and a through lens detector (TLD) for high magnification images were used for the collection of SE images.

### 2.7 SEHI acquisition and image processing

The FEI Nova Nano 450 SEM is provided with a through lens detector which includes a voltage controlled deflector electrode. The deflector electrode channels the signal into the SE detector. The deflector electrode is set to predetermined deflector voltages and an image is generated for each deflector voltage. Spectra and hyperspectral images are acquired through post-processing of such image series. Stage bias experiments have been performed to allow energy calibration of this process through experiments. Fiji software was utilised to perform image processing and extract S-curves from the SEHI stacks. Histogram and spectral off-set normalisation were applied retrospectively to optimise all images for brightness and contrast, and to limit the effects of differing sample work functions. Once S-curves were obtained they are differentiated to produce the SE spectra. Component analysis was then preformed to image stacks utilising non-negative matrix factorization (nnmf) to isolate components of interest.

### 2.8 Statistical analysis

Statistical analysis was performed using the GraphPad Prism version 9 software (GraphPad Software, Inc.; La Jolla, USA). Based on One-Way ANOVA nonparametric analysis, the Kruskal–Wallis test followed by the Dunn's post hoc test for multiple comparisons was used for data that was not normally distributed. Data are reported as mean ± standard deviation. The significance level was defined as *p* < 0.05.

Detailed methodology for High Temp GPC and XPS is given in the ESI.†

## 3. Results

### 3.1 Effect of oxidative stress and dynamic distention on the bulk mechanical properties of the PP mesh

The tensile properties of Gynemesh and Restorelle were measured using a tensiometer and comparisons made to control samples which were placed in the bioreactor with dH_2_O (distilled water) but without any strain forces (see Fig. 2). The results displayed in Fig. 2 show the bulk mechanical properties of both mesh materials were affected by dynamic distention. After dynamic distension in dH_2_O for 6 h, both Gynemesh and Restorelle exhibited a steeper gradient in the average stress–strain curves than that of the control non-stretched material. A similar finding is observed when both dynamic distention and oxidative stress are combined, particularly for Gynemesh; however, this is not so clear for Restorelle as the stress–strain curves collected show large variations; which is especially noticeable at higher strains (>40%). These variations are also observed for Restorelle when the highest dynamic distention is applied (50% 6 hours in dH_2_O). However, oxidative stress on its own did not affect the bulk mechanical properties of either material, but there was a large degree of variability for Restorelle with large variations particularly at higher strains (>40%).

**Fig. 3:**
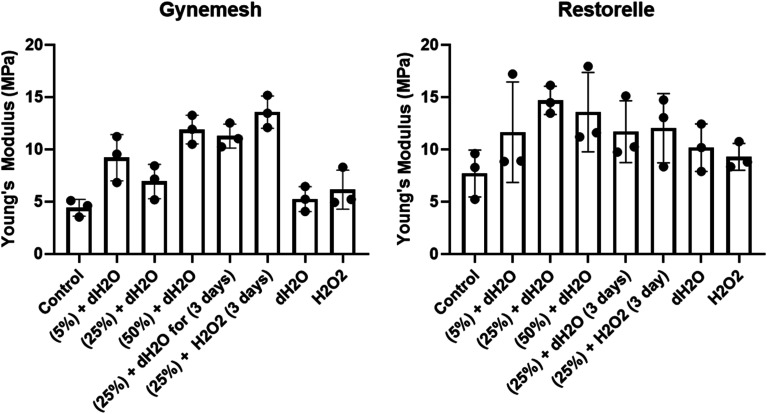
Young's modulus of Gynemesh (left) and Restorelle (right) after; 5%, 25% and 50% dynamic distention for 6 hours, 25% dynamic distention within dH_2_O or H_2_O_2_ for 3 days, and 14 days incubation at 60 °C within dH_2_O or H_2_O_2_ in comparison to that of the control material. Mean ± SD (*N* = 3).

The Young's modulus of each sample was determined from the stress–strain curves of the tested materials compared to that of their controls (Fig. 3). Gynemesh showed a significant increase in the Young's modulus compared to the control for the samples subjected to 50% dynamic distention for 6 hours and to 25% dynamic distention with H_2_O_2_ for 3 days. There were similar trends observable for Restorelle, however these differences did not meet statistical significance as a result of high variability between samples. The Young's modulus of samples only exposed to oxidative stress was similar to the control.

### 3.2 Effect of oxidative stress and dynamic distention on the surface and bulk molecular structure of PP mesh

**Fig. 4:**
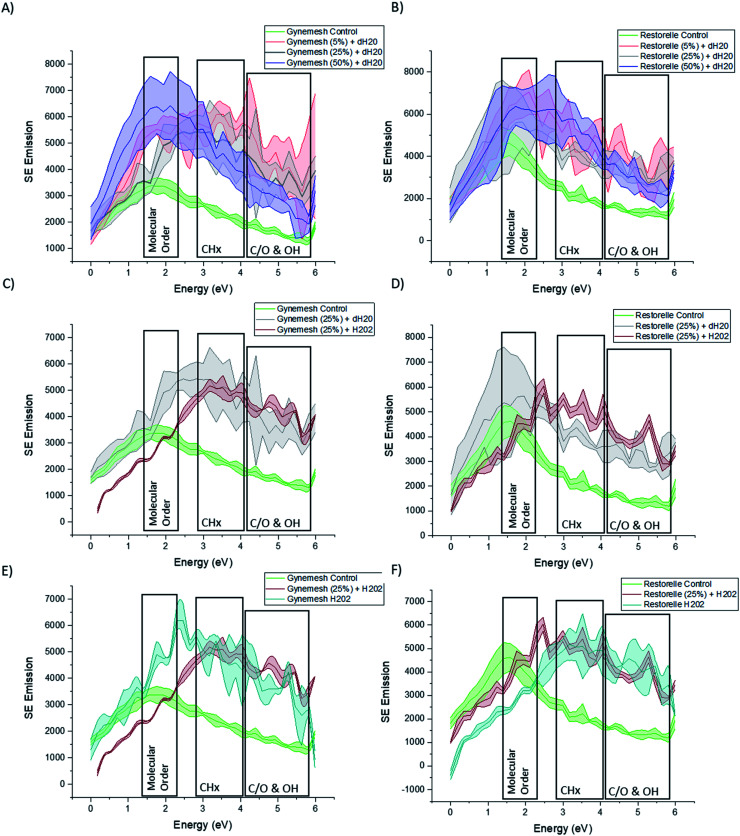
Secondary electron spectra for Gynemesh (*n* = 4) (A), (C) and (E) and Restorelle (*n* = 4) (B), (D) and (F) after dynamic distention at varying degrees (A) and (B), after treatment combining 25% dynamic distention with H_2_O_2_ (C) and (D) and after treatment with H_2_O_2_ alone (E) and (F).

Fig. 4A and B shows the SE spectra of Gynemesh and Restorelle after 5%, 25% and 50% dynamic distention compared to that of control non-treated materials. Within the collected SE spectra for all conditions of both Gynemesh and Restorelle, some differences in intensities within the 1.4–2.3 eV range are noticeable. Previous studies have isolated the energy range of 1.4–2.3 eV to the molecular order/crystallinity of polymers.^19,21,22^ As the spectra in Fig. 4A and B display an increased intensity in this energy range compared to the respective control, either the molecular order/or crystallinity at the mesh surface are increased as a result of the distention testing. There is a larger variation in surface molecular order/or crystallinity as a result of distention testing amongst the analysed areas. This is reflected in the increased width of errors bands in the spectra compared to the spectra collected from the respective control surfaces. Such large intensity increase in the 1.4–2.3 eV range is present for dynamic distention as low as 5% after testing in dH_2_O (Fig. 4A and B). SE emission peaks are also present within the ranges of 2.7–4.3 eV. This energy range has been routinely associated with (CH_*x*_).^18,21,27^ When comparing test samples with that of their controls it is notable that each test sample surface exhibits an increase in this energy range.

Fig. 4C and D presents the SE spectra of both Gynemesh and Restorelle after 25% dynamic distention when combined with 3% H_2_O_2_ treatment or dH_2_O, respectively. The exposure to 3% H_2_O_2_ during mechanical testing resulted in marked differences compared to testing in dH_2_O. Both Gynemesh and Restorelle surfaces repeatedly mechanically loaded and unloaded within the presence of H_2_O_2_ are more similar to the SE spectra of their controls in the energy range associated with molecular order/crystallinity than surfaces exposed only to dH_2_O during distention testing. This is most prominent within Restorelle after 25% dynamic distention in the presence of H_2_O_2_. However, the emission in the energy range associated with CH_*x*_ bonding is still increased compared to the control. Bouhelal *et al.*^25^ used FTIR to characterise an increase in CH_2_/sp^2^ bonding which was related to the formation of ethylenic chain crosslinks in the presence of Peroxide. Results obtained from both SEHI and tensile testing further support previously reported findings which state that peroxide-initiated crosslinking causes the formation of PP crosslinks which in combination with the highlighted molecular order variations, is a fundamental driver of the changes observed within tensile properties of Gynemesh and Restorelle after 25% dynamic distention in the presence of H_2_O_2_.

SE spectra provided in Fig. 4E and F show how the effect of exposure to H_2_O_2_ treatment alone reduces error bands in molecular order SE region. H_2_O_2_ treatment makes PP surfaces more homogeneous in terms of molecular order, however it still introduces inhomogeneity in terms of CH_*x*_ and CO SE emissions. In brief SE spectra show that dynamic distention alone causes surface molecular order variability to increase, however, H_2_O_2_ alone causes molecular order variability to reduce. These findings provide clear evidence that surface molecular order is affected by the testing environment.

SEHI provides information on the superficial surface (only a few nanometres deep) of the material. To assess if related molecular changes can be seen in the bulk material high temp GPC was performed on Gynemesh samples (data provided in the ESI†). It was noted that when compared to the control, Gynemesh samples undergoing mechanical distention alone and also when combined with 3% H_2_O_2_ treatment showed an increase in molecular weight. The high temp GPC data alongside SE spectra further supports the hypothesis that both mechanical distention and oxidation (experimentally achieved through the addition of H_2_O_2_) trigger molecular structure changes within Gynemesh and Restorelle both at the surface and within the bulk.

### 3.3 Effect of oxidative stress and dynamic distention on the surface and bulk oxidation of PP mesh

To consider the effects of oxidative stress and dynamic distention causing oxidation on both Restorelle and Gynemesh, SE spectra and X-ray photoelectron spectroscopy (XPS) data were collected. Fig. 4 displays SE spectra measurements of functional groups linked as oxidation products (C/O & OH) (4.2–6 eV). Earlier studies have provided confirmation that this SE energy range is associated with CO, OH and COO bonding.^21,22^ In response to mechanical distention alone (5%, 25% and 50%) SE spectra obtained from both Gynemesh and Restorelle (Fig. 4A–D) under dynamic distention in the presence of H_2_O and H_2_O_2_ exhibit an increase in SE emission associated with an increase in surface oxidation. When both materials are subject to H_2_O_2_ treatment alone with no dynamic distention oxidation SE peaks are still prominent (Fig. 4E and F). The largest increase in surface oxidation for Restorelle is observed when H_2_O_2_ is present with or without dynamic distention. In contrast the Gynemesh surfaces do not exhibit such a clear increase in oxidation products resulting from the presence of H_2_O_2_ during dynamic distention. In fact, at some energies within SE emissions associated with the presence of oxygen there is a reduction in emission. To corroborate these results XPS data is provided in Fig. 6A which shows an increase in O/C ratio on the surface of Gynemesh as a result of mechanical distention.

**Fig. 5:**
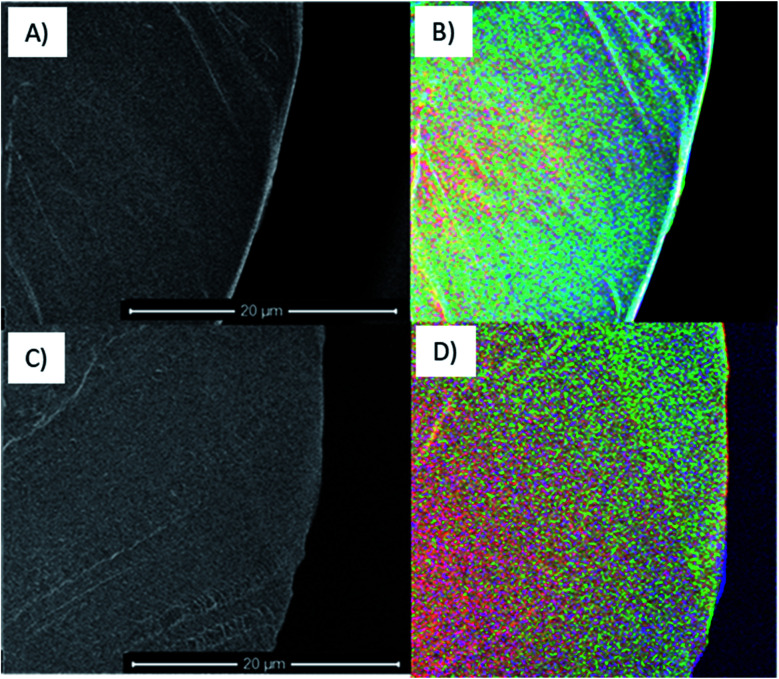
SEHI images generated from automated SEHI colouring of cross sectioned Gynemesh after 25% mechanical distention with 3% H_2_O_2_ treatment (A) and (B) and Gynemesh – no treatment (C) and (D). Red regions symbolise high molecular order, green regions symbolize CH_*x*_, and blue region indicate (CO, COO, OH) oxidation products.

**Fig. 6:**
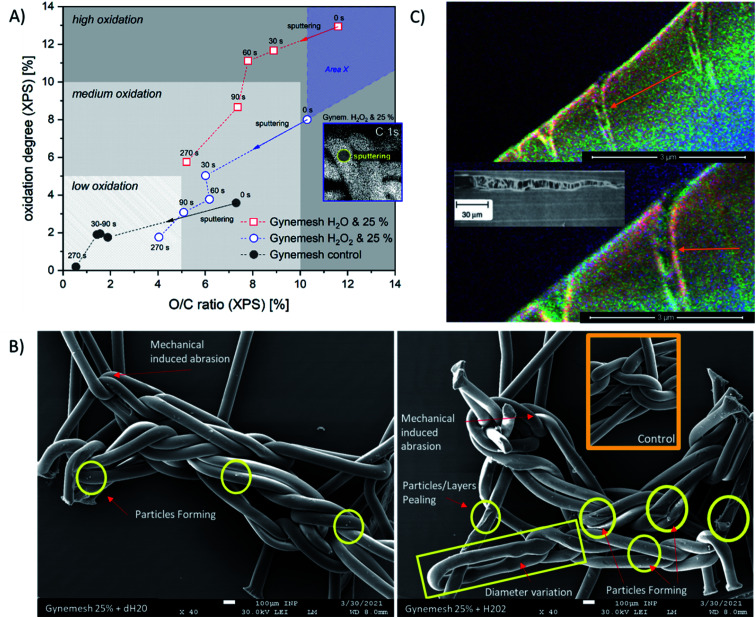
(A) Oxidation of Gynemesh samples characterized by means of XPS depth profiling (sputter time in seconds (s)), three levels of oxidation are indicated by step of 5% change, area *X* indicates potentially higher oxidation of the samples before the analysis, inset: XPS image of C 1s for Gynemesh after 25% mechanical distention with 3% H_2_O_2_ treatment. (B) SE images of Gynemesh after 25% mechanical distention with 3% H_2_O_2_ treatment, Gynemesh after 25% mechanical distention and inset SE image of control non treated Gynemesh. (C) SEHI images generated from automated SEHI colouring of Gynemesh after 25% mechanical distention with 3% H_2_O_2_ treatment. SEHI image shows formation of cracks of the Gynemesh after treatment.

Fig. 5 shows SEM (A) and (C) and SEHI (B) and (D) maps obtained from cross-sections of control Gynemesh (C) and (D), and Gynemesh after 25% mechanical distention with 3% H_2_O_2_ treatment (A) and (B). SEHI maps presented in Fig. 5 shows the depth of oxidation encroachment into the Gynemesh fibres after this accelerated degradation and dynamic distention (image B).

The colour regions highlight the response to different chemical functional groups. The SEHI map shown in Fig. 5B visualises the presence of oxidation products (OH, CO, COO) (in blue) even deep within the 25% H_2_O_2_ Gynemesh in areas where the original surface is preserved (*e.g.* no peeling has occurred), whereas for the Gynemesh control sample in Fig. 5D oxidation is visible only near the surface. To corroborate these findings XPS analysis was applied to the same two samples to generate a similar oxidation depth profile. In this process an argon cluster beam was used to sputter the material for 30 s before being turned off whilst XPS spectra were acquired. Successively, 4 levels of a depth profile were achieved by sputtering with the cluster beam.

The XPS depth profiling (longer sputter time (s) corresponds to analytical signal from greater depth) shown in Fig. 6A demonstrates the oxidation pathway from surface into the material displayed using two oxidation parameters: relative oxygen concentration (O/C) and oxidation degree (sum of C–O, C=O, and COO binding related to C–C). Here, the O/C ratio correlates with the oxidation degree of PP in all samples positively. These XPS results reveal that oxidation is occurring in all samples, with the highest levels of oxidation at the surface (sputter time 0). As sputter time is increased the oxidation level for all materials is reduced. All treated and/or loaded samples are more oxidized than the control sample. Both XPS and SEHI show that when dynamic distention is combined with exposure to H_2_O_2_ the O/C ratio at the Gynemesh surfaces is lower than when testing was carried out in dH_2_O. We assume this is in response to the combination of sample stretching and oxidation by H_2_O_2_ causing chemically derived surface chain scission resulting in surface fragmentation being cleaved from the surface as a response. In Fig. 6A we introduce “area *X*” – an area of the material with fragments of the Gynemesh sample treated with H_2_O_2_ and exposed to mechanical load with 25% stretching in order to underline the effect. In the area *X* oxidised fragments of the original surface are detected when they stick fully to the fibres. However, due to the partial detachment of oxidized fragments, deeper layers of fibres can be uncovered at any point, which can be less oxidized than intact samples or other intact areas. The indication of this effect is given by SE images (Fig. 6B), which demonstrates several defects after sample loading and peroxide treatment. The level of oxidation seen deeper within the fibre after distension testing at 25% dynamic distention exceeds the surface oxidation present in the control sample further confirming the SEHI image results.

A potential mechanism for the deep reaching oxidation after distension was revealed by the capture of the SEHI images presented in Fig. 6C that may provide an insight into the mechanism that enables oxidation to penetrate the material. These images reveal cracks of a PP fibre perpendicular to the direction of tensile stress caused by 25% distention and exposure to H_2_O_2_.

### 3.4 Detecting insoluble oxidation products from PP mesh after oxidative stress and dynamic distention conditions

Findings within high temp GPC data (provided in ESI†) and XPS (Fig. 6A) showed that combining both mechanical distention with H_2_O_2_ treatment produced an increase in PP surface fragments. The visualisation of these fragments is highlighted in Fig. 6B where the prevalence of particles is greater on the surface of PP subject to 25% mechanical distention with H_2_O_2_ treatment compared to distention with dH_2_O. To identify the composition of these products conventional SEM imaging with SE spectra collection was performed on 25% mechanical distention with H_2_O_2_ treated samples.

**Fig. 7:**
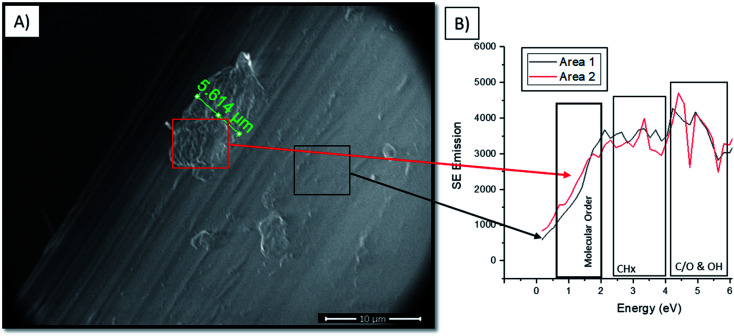
(A) SEM images of the surface of Gynemesh after 25% mechanical distention with 3% H_2_O_2_ treatment. Image shows material degradation of the surface of the material. (B) Secondary electron (SE) spectra for Gynemesh after 25% mechanical distention with 3% H_2_O_2_ treatment, with SE spectra taken from two regions of the surface; red area region showing strong emission in molecular order range (0–1.4 eV) when compared to that of the black area region whose emissions are consistent with the typical surface emissions of the material.

Fig. 7A shows the formation of the highly crosslinked & highly oxidized insoluble regions on the Gynemesh surface (after 25% dynamic distention with H_2_O_2_). Fig. 7B displays the SE spectral region associated with molecular order and identifies the area 2 (the surface fragment) to be a higher order compared to the surrounding Gynemesh surface. These results compare closely to the SE spectra presented in Fig. 4E which also indicated that the general surface of PP treated with 25% mechanical distention with H_2_O_2_ treatment to exhibit a greater amount of crosslinking and surface oxidation than that of control PP.

## 4. Discussion

### 4.1 Novelty and significance

**Fig. 8:**
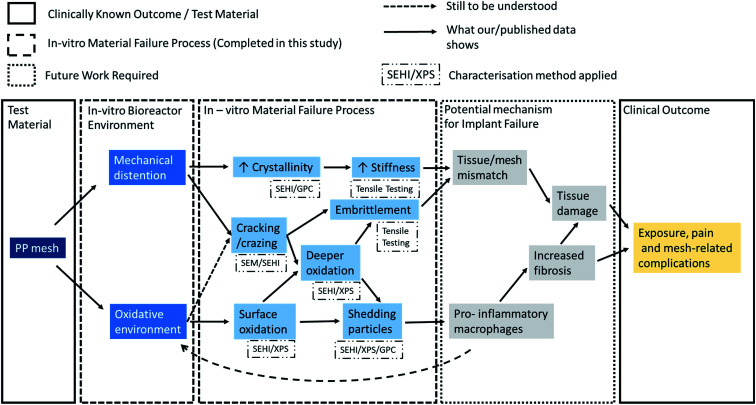
Schematic of study experimental steps and findings.

To date it is not clear, whether vaginal PP mesh failure results from bulk or surface functional failures or if failure is caused by a combination of both. Insights into the failure mechanisms are needed to either remedy the problems with current materials (*e.g.*, by surface treatments of existing materials prior to implantation) or to inform design and pre-implantation testing of new materials. Pre-implantation screening tests should be able to replicate the chemical environments and the repeated mechanical energy input experienced by meshes implanted into the pelvic floor environment.^28^ This study demonstrates the value of using SEHI to detect and characterise localised surface changes to materials in combination with established bulk analysis techniques. Fig. 8 summarises at a glance the potential mechanisms responsible of the clinical complications observed with these meshes based on previous hypothesis and further supported by the data reported in this study.

### 4.2 Aim of this study

Our aim in this study was to critically evaluate how mechanical distention and an oxidative environment affect the fundamental physical properties of PP mesh, to understand whether PP materials degrade and change post implantation and whether could this affect the host response to these materials. This led to a series of research questions:

• Is there any evidence that oxidative stress can alter PP fibre surfaces?

• Is there any evidence that mechanical distension can alter PP fibre surfaces?

• Is there any evidence of an additive effect and if so what is the impact of oxidative stress and of mechanical distension on PP fibre surfaces?

• Is there any evidence of PP fibres producing surface particles in response to oxidative or mechanical stress which may detach from the surface?

### 4.3 The main findings of this study and relation to the previous literature

#### 4.3.1 Evidence that oxidative stress can alter PP fibre surfaces?

Our study utilising the surface sensitive SE spectroscopy revealed increased surface oxidation on all tested materials compared to that of the control samples. This finding was corroborated by XPS, a well-established surface analysis tool. It has been long observed that even a little oxidation can result in stress-cracking in semi-crystalline polyolefin, with embrittlement observed in PP.^29,30^ By applying SEHI to cross-sections it was further established from SEHI maps (Fig. 5) that after testing in H_2_O_2_, oxidation can be present several micrometres deep at a level that exceeds that of the surface oxidation found in the control sample. The high level of oxygen, after testing, found beneath the surface was also confirmed by XPS (Fig. 6A). Previous studies analysing PP mesh samples explanted from patients or animal models provide evidence of oxidative degradation through the appearance of hydroxyl and carbonyl groups in the material.^8,14,31–33^ However, the results of these explant studies have been challenged because of the use of fixatives, including formalin on the samples prior to analysis. Formalin is capable of causing surface changes which could result in the presence of oxygen containing functional groups. Formalin has also been shown to initiate the PP surface cracking.^34^ To remove the chance of any formalin-material induced surface changes this study obtained samples without the use of any fixatives or sample treatment and therefore supports the view that an oxidative environment could very likely cause the observed surface oxidation.

An alternative explanation for the presence of oxygen as confirmed by XPS is the absorption of dH_2_O/H_2_O_2_ into PP. The maximum reported dH_2_O content is 0.1 wt%.^35^ The saturation concentration of H_2_O_2_ of PP immersed in H_2_O_2_ at 35 °C was reported to be ∼1.2 × 10^−2^ kmol m^−3^.^36^ However, as both SEHI and XPS show an increase in oxygen when any level of distention is applied, compared to the control sample (immersed but without distention), other oxygen containing species must be a result of the distention. Interestingly, the presence of the oxidising environment of H_2_O_2_ seems to have a smaller effect on the generation of oxygen containing groups than the application of distention. In fact, the nature of the oxidising environment seems to be altered by the mechanical distention.

#### 4.3.2 Evidence that mechanical distension can alter PP fibres?

As pointed out in 4.1 fibres must be considered for their (i) bulk and (ii) surface functions before outlining (iii) mechanism for their alteration.

(i) Surface properties: SE allows one to see the effects of dynamic distention on the surface molecular structure of Gynemesh and Restorelle. Collected SE spectra/GPC results displayed changes in molecular order for all materials after mechanical testing compared to that of the control samples. For Gynemesh it was seen that there is an increase in surface molecular order which occurs with greater dynamic distention when mechanical testing is carried out in dH_2_O. In this environment the mechanical stresses are giving rise to the formation of significantly more ordered fibres surfaces, which is not the case when H_2_O_2_ is present during mechanical testing although other chemical changes (CH_*x*_ bonding O/C ratio) are still observed as explained in Section 3.2. These results leave no doubt that mechanical distention does alter the fibre surface. In addition, our findings strongly suggest that the mechanical distention in the correct environment is essential in order to see representative change to the fibre surfaces resulting from the mechanical testing.

(ii) Bulk properties: while it is well known that surface flaws can be responsible for the initiation of mechanical failure, the effect of such initiation sites on the mechanical performance will depend on the bulk material properties. In this respect, we note that the short testing time of only 6 h seems to be well suited to observe the early stages of failure initiation and their effect on the bulk mechanical properties, even at relatively low dynamic distention. Both Gynemesh and Restorelle are susceptible to the formation of more ordered fibres when tested using 5% distention in dH_2_O with changes in CH bonding and O/C ratio. Even during this short test duration, the surface changes are reflected in the mechanical data presented which also shows a similar trend; *e.g.* as dynamic distention is increased the stiffness of the Gynemesh increases. While this might be surprising there is a simple explanation for this effect.

(iii) Mechanism for the observed surface/bulk alternations: the degree of distention affects the quantity of oxygen containing species (see 4.5.1), pointing to the production of oxygen containing species as a result of the periodic mechanical energy input. Mechanical energy can lead to bond breaking.^48^ For polypropylene in particular it has been shown that the localised bond breaking leads to a strong increase in oxygen containing end groups through chain scission.^37^ The effect was found to be stronger at the surface, compared to the bulk, consistent with our findings and explained as a result of surface bonds being exposed to higher local loads and higher concentrations of oxygen. The formation of new chemical groups as a result of mechanical energy input is known as mechano–chemistry. In addition to the production of radicals, changes in molecular weight (MW) and molecular order are a hall mark of mechano–chemistry. In solid materials the result of mechano–chemical reactions is the local built up of radicals. Depending on radical concentrations the MW/molecular order can locally increase (at high concentrations) until crosslinking occurs or decrease (at low radical concentrations).^38^ This dependence explains the presence of localised highly crosslinked areas as found in Fig. 7 that alter the surface topography locally. Another local topography altering effect in response to distention is that of crazing as reported in Fig. 6C. This leads to a local crystallinity increase at the surface, which could alter the strains experienced in the bulk, thus local bulk mechano–chemistry. Therefore, it is important to highlight that mechano–chemistry products for potential new implants must be considered, as they alter surface and bulk chemistry as well as surface topography. Many of these changes are hard to predict as differences in local energy input during mechanical testing can have significant effects, as explained further below.

Consideration of the mechano–chemical reactions provide insights as to why the results of Gynemesh and Restorable from XPS, GPC and tensile testing results differ in this study.

**Fig. 9:**
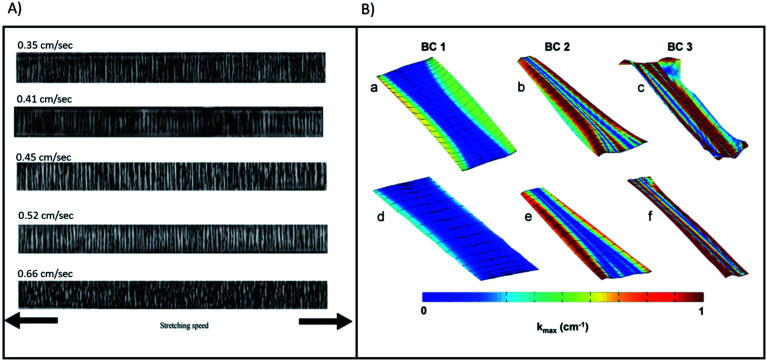
(A) (a)–(e) presents transmission optical micrographs for samples of PP with stretching speed values 0.35, 0.41, 0.45, 0.52 and 0.66 cm s^−1^, respectively. Reproduced and rescaled from^39^ under the Creative Commons Attribution International License (CC BY) http://creativecommons.org/licenses/by/4.0/. (B) Contour map of |*k*_max_| for representative Gynemesh (a)–(c) and Restorelle (d)–(f) samples at 10 N. Boundary conditions (BCs) 1, 2, and 3 are represented by (a) and (d), (b) and (e), and (c) and (f) respectively. Solid black lines represent the direction of *k*_max_. Reprinted from^40^ with permission from Elsevier.

Most notable were the changes in tensile properties after dynamic distention. The rationale for the differences observed between the meshes can be explained through the results of previous studies (highlighted in Fig. 9) and the mechanism of mechano–chemistry discussed previously. Mechano–chemistry reactions require the input of kinetic energy,^48^ provided in this study *via* dynamic distention (related mechanical strain). This energy causes the molecular chain extensions which produce the radicals required for chain oxidation, molecular alignment and consequently fibre stiffness.^37,38^ The SEHI results show both oxidation of PP and molecular order differences with large variations across both materials surface. Therefore, the driving factor behind them must be localised and non-uniform across the samples. The reason for this is proposed to be due to the different abilities of the two different meshes to manage strain related pressures whilst the material undergoes dynamic distention. Fig. 9A shows how a single fibre of PP stretches, deforming, creating cracks and crazes. Similar crazing was observed in this study with SEHI images identifying “crazes” on the surface of a PP fibre after 25% distention and exposure to H_2_O_2_. Of interest within the images provided is how the single fibres deform non-uniformly and produce crazes or cracks in different places across the PP fibre. When considering a mesh as a woven structure, Fig. 9B shows Gynemesh and Restorelle experience different strains locally whilst undergoing uniaxial flexion corroborating the findings within this study.

Fig. 9B visualises non-homogeneities in curvature distribution of both Gynemesh and Restorelle. This study observed the greater ability of Restorelle to cope with strain induced stresses as a result of its knitted geometry.^41^ Such findings further highlight the requirement for greater consideration to be given to the mechano–chemistry of future implants which also undergo similar non-uniform strain. Having a better understanding of how an implant responds to mechanical strains, would allow a deeper insight into the mechano–chemical interactions occurring within the material whilst under stress-corrosion.

The mechanism by which PP meshes fail post implantation is still open to debate, evidence has been presented that points to environmental oxidation degradation (*i.e.* stress-corrosion) as a potential option but the published literature is not consistent on this point. The problem is the absence of conclusive experimental results to support the *in vivo* failure mechanism taking account of the fact that surface and bulk morphology are affected by mechanical forces that undoubtedly vary from patient to patient.

#### 4.3.3 Is there an additive effect of oxidative stress and of mechanical distension on the PP mesh?

Due to the highly localised nature of surface modifications any additive effects are also highly localised. As a result, in some areas there will be no additive effects, whereas in other areas there can be strong additive effects. Such local variation does impact the mechanical properties at least in the distention tests carried out in this study as evidenced by the large error bands in stress–strain curves at high strains obtained after prolonged testing in Fig. 2. In the context of mesh implants, the presence of locally varying acceleration factors (mechanical load, mesh extension, oxidative environment *etc.*) is highly likely to impact the structural shape of the mesh. The resulting local stiffness increases can have a negative impact if the mechanical properties of the material locally diverge strongly from those of the softer more pliable native tissue,^8^ a consequence which potentially increases the likelihood of possible complications.^42^ For instance, local surface structural variations were found in this study through crazes and cracking. In this study, cracking of the PP is due to two factors: tensile forces and chemical degradation of the material by oxidizing substances. Both factors reinforce each other. The first cracks in the material create a new contact surface for oxidizing agents, which can then penetrate faster and deeper into the material and further weaken its strength (as given in Fig. 8). Thus, such a combined mechanism could turn out to be a significant driver of host tissue damage.

Peroxides are radical initiators and H_2_O_2_ is a commonly used source of oxygen-derived free radicals. As highlighted in Fig. 8 it is therefore reasonable to assume H_2_O_2_ plays a role in chain scission on the surface of the material which results in the formation of oxygen containing compounds. From the tensile test data provided it was shown that both meshes undergoing dynamic distention in the presence of H_2_O_2_ were noticeably stiffer at low strains than that of samples immersed in dH_2_O. The reasoning behind this could be explained by the mechano–chemical reactions described in 4.5.2. With the greater presence of radical initiators provided by the enhanced H_2_O_2_ ingress in crazed areas, the threshold required to induce mechano–chemical radical formation in the bulk can be breeched, leading to the creation of local regions stiffer in the bulk. This was highlighted by GPC findings showing an increase in MW post H_2_O_2_ and dynamic distention. How this translates into the stiffness of the mesh will depend on the mesh geometry and is likely to be the cause of the large variations in the stress–strain curves at higher strain observed in 3.1 as different locations will fail at different applied strains. Aside from this, tensile data also indicated that post H_2_O_2_ exposure and distention overall the material became slightly more ductile. This can be explained through SEHI surface data which showed CH_*x*_ bonding within H_2_O_2_ treated materials increased when compared to that of the controls. PP crosslinking instigation can have a multitude of mechanisms depending on the reaction conditions, however, as previously highlighted ethylenic chain crosslinks form in the presence of peroxide which has also been shown to increase the ductile facture properties of PP.^25,43^

As shown in Fig. 8, the consequence of producing a stiffer material is expected to result in a tissue/mesh mechanical property mismatch. Critically, an increase in vaginal tissue degradation has been associated with the use of mesh of increased stiffness and higher weight.^42^ Even if there is no overall mismatch when comparing the mechanical properties of the whole mesh this would not exclude the presence of a large localised mismatch in areas subject to additive effects. When assessing the combined impact on patient outcome, one cannot ignore changes in surface topography as the topography has shown to impact macrophage polarisation.^44^ To what extent this form of failure impacts tissue fibrosis or general tissue damage is yet to be fully investigated.

#### 4.3.4 Is there any evidence of PP fibres producing surface particles in response to oxidative or mechanical stress which may detach from the surface?

Previous studies have indicated that an oxidising environment can degrade PP.^31^ However, PP's chemical structure does not contain any chemical functionality that is expected to bear oxygen. Yet, in response to mechanical distention alone (5%, 25% and 50%) both SEHI and XPS indicated an increase in oxidation products at the surface of both meshes. When substituting dH_2_O with H_2_O_2_ both SEHI and XPS also showed oxidation of the materials was notably increased. High temp GPC data showed that combining mechanical distention with H_2_O_2_ treatment produced an increase in insoluble products. It is thought that these products consist of highly cross-linked regions of PP which may be “etched” away as surface oxidation causes molecular chain scission. Conventional SEM imaging alongside SE spectra collection was undertaken to see if it was possible to identify insoluble oxidation products released from the PP mesh. From the SEM analysis conducted it appears that the highly cross-linked structures shown in Fig. 6, are not uniform across the samples surface. This again indicates that there is no visual uniform failure, but highly localised random material degradation, most likely as a result of mechano–chemistry. Therefore, to evaluate this surface degradation a spatially resolved chemical analysis capability is required. The advantage of using SEHI over traditional surface analysis methods is that local variations in chemical alterations occurring at the nanoscale can be observed, variations which can be hidden in large area average surface analysis techniques. Many of the averaging techniques used in previous studies are not able to reveal the onset of the degradation on the surface which occurs over a few microns.

The highly cross-linked/highly oxidized PP which were observed forming on the mesh surface were insoluble by GPC. We suggest that as the dynamic distention force increases, oxide products are likely to be expelled from the surface mesh fibres and are therefore no longer visible within the SE spectra. While no study to date has actively searched for degraded PP, despite it being highlighted as an area of interest previously,^45^ the strongest evidence for *in vivo* PP degradation was the discovery of the presence of blue granules (thought to be from the dye introduced to Gynemesh fibres) from a histological section of an explanted Ethicon transvaginal mesh.^8^ Previous studies exploring oxidation of PP have shown many different routes for the oxidation of PP and revealed that the resultant oxidation products can vary from harmless to toxic in their effect on human tissue cells.^46^ Previous studies have also indicated uncoated polypropylene mesh can elicit a dominant M1 macrophage response at a PP mesh fibre surface.^47^ The specific macrophage activation will determine the healing process, and an M1 macrophage phenotype has been associated with a chronic immune response and rejection, leading to an increased formation of fibrotic tissue.^6^ We suggest it is feasible that the PP oxidation products released into the *in vivo* mesh environment may instigate the inflammation of nearby cells which in turn release more oxidizing agents. Recent studies focused on the toxicology of PP material have shown that PP alone has the potential to trigger an increase in cell inflammation which in turn leads to an increase in PP fibre oxidation products.^46^

### 4.4 Future work implications for pelvic floor biomaterials – a multi-factorial problem?

From the data provided it is possible to propose a mechanism that potentially describes how such meshes might fail clinically, and future work will be needed to confirm the mechanisms proposed in Fig. 8. While there are general risk factors (obesity and oestrogen status) clinical complications can be patient specific and most complications occur in a time range of one to five years after the operation. Studies have also shown that the PP mesh materials removed post implantation exhibit an increase in stiffness and serve surface deformation (6). Therefore, it may be postulated that the implanted mesh material within these patients may have passed the dynamic distention threshold at least locally for mechano–chemistry reactions to result in local increased stiffness. This raises the question of why does clinical failure of the PP mesh only occur within a subset of patients and why mesh material stiffening apparently leads to tissue inflammation?

The results provided in this study offer an explanation based on the mechano–chemistry reaction to which the PP fibres are subjected to clinically. Patients who require the implantation of a pelvic floor mesh are likely to each express an individual profile of mechanical stresses onto the implanted mesh which in turn may elicit a different response from the mesh. It can be seen that the greater the stresses applied on the implant the greater the mechano–chemical reaction will be, resulting in the formation of more radicals and greater material stiffness. This in-turn would produce more oxidised PP particles which increases the likelihood of a harmful inflammatory response. However, to understand this relationship requires the consideration of two forms of oxidation, surface and bulk, both of which are different mechanisms of oxidation. From the data provided in this study, surface oxidation is essentially a form of chemical etching with bulk oxidation occurring in response to mechano–chemistry free radial formation. The impact of both mechanisms will depend on the local mechanical load affecting the PP mesh. Previous work has been successful in identifying oxidation products and material stiffness, as highlighted in this study, but with the prior use of only bulk analytical chemical averaging methods they have not been able to establish that the process hinges on localised mechanical stress related processes.

## 5. Conclusions

This study introduced a simple novel *in vitro* test method which has the potential of evaluating future PP mesh materials for clinical suitability without the requirement for initial *in vivo* studies. The test approach included mechanical distension combined with hydrogen peroxide exposure to mimic the conditions of irregular distension and oxidative environment to which all materials implanted in the vagina are exposed. A key benefit of the test method chosen was the avoidance of the use of any fixative treatment, removing concern regarding the introduction of contamination artefacts as claimed in previous studies.

In addition to the mechanical test data provided, this paper has presented key insights into the mechano–chemistry reaction of PP causing, polymer oxidation, changes in molecular structure, crack/craze formation and the release of etched oxidised insoluble particles. SEHI, a technique providing high resolution surface chemical mapping provided for the first time a route to link the effect of localised stresses to reactions of mechano–chemistry within PP. The method of mechanical distension testing during hydrogen peroxide exposure followed SEHI image analysis could form the basis of an “early warning” system which possesses the ability to identify materials which are not appropriate for clinical deployment within a hostile *in vivo* environment.

## Conflicts of interest

There are no conflicts to declare.

## Supplementary Material

RA-011-D1RA05944K-s001

## References

[cit1] Mangir N., Aldemir Dikici B., Chapple C. R., MacNeil S. (2019). Nat. Rev. Urol..

[cit2] Roman S., Mangir N., Bissoli J., Chapple C. R., MacNeil S. (2016). J. Biomater. Appl..

[cit3] Barski D., Deng D. Y. (2015). BioMed Res. Int..

[cit4] Artsen A. M., Liang R., Meyn L., Rytel M., Palcsey S., Abramowitch S. D., Moalli P. A. (2020). Acta Biomater..

[cit5] Osman N. I., Roman S., Bullock A. J., Chapple C. R., MacNeil S. (2014). Proc. Inst. Mech. Eng., Part H.

[cit6] Nolfi A. L., Brown B. N., Liang R., Palcsey S. L., Bonidie M. J., Abramowitch S. D., Moalli P. A. (2016). Am. J. Obstet. Gynecol..

[cit7] Roman S., Mangir N., MacNeil S. (2019). Curr. Opin. Urol..

[cit8] Imel A., Malmgren T., Dadmun M., Gido S., Mays J. (2015). Biomaterials.

[cit9] Talley A. D., Rogers B. R., Iakovlev V., Dunn R. F., Guelcher S. A. (2017). J. Biomater. Sci., Polym. Ed..

[cit10] Kurtz J., Rael B., Lerma J., Wright C., Khraishi T., Auyang E. D. (2016). Surgical Endoscopy.

[cit11] Wood A. J., Cozad M. J., Grant D. A., Ostdiek A. M., Bachman S. L., Grant S. A. (2013). J. Mater. Sci.: Mater. Med..

[cit12] Klink C. D., Junge K., Binnebosel M., Alizai H. P., Otto J., Neumann U. P., Klinge U. (2011). Journal of Investigative Surgery.

[cit13] Iakovlev V. V., Guelcher S. A., Bendavid R. (2017). J. Biomed. Mater. Res., Part B.

[cit14] Costello C. R., Bachman S. L., Ramshaw B. J., Grant S. A. (2007). J. Biomed. Mater. Res., Part B.

[cit15] Thames S. F., White J. B., Ong K. L. (2017). International Urogynecology Journal.

[cit16] Kazemian P. S., Mentink A. M., Rodenburg C., Humphreys C. J. (2007). Ultramicroscopy.

[cit17] Stehling N. A., Masters R., Zhou Y., O'Connell R., Holland C., Zhang H., Rodenburg C. (2018). MRS Commun..

[cit18] Abrams K. J., Dapor M., Stehling N., Azzolini M., Kyle S. J., Schäfer J. S., Quade A., Mika F., Kratky S., Pokorna Z., Konvalina I., Mehta D., Black K., Rodenburg C. (2019). Adv. Sci..

[cit19] Masters R. C., Stehling N., Abrams K. J., Kumar V., Azzolini M., Pugno N. M., Dapor M., Huber A., Schäfer P., Lidzey D. G., Rodenburg C. (2019). Adv. Sci..

[cit20] Wan Q., Abrams K. J., Masters R. C., Talari A. C. S., Rehman I. U., Claeyssens F., Holland C., Rodenburg C. (2017). Adv. Mater..

[cit21] Farr N., Thanarak J., Schafer J., Quade A., Claeyssens F., Green N., Rodenburg C. (2021). Adv. Sci..

[cit22] Farr N. T. H., Hamad S. F., Gray E., Magazzeni C. M., Longman F., Armstrong D. E. J., Foreman J. P., Claeyssens F., Green N. H., Rodenburg C. (2021). Polym. Chem..

[cit23] Roman S., Mangir N., Hympanova L., Chapple C. R., Deprest J., MacNeil S. (2019). Neurourol. Urodyn..

[cit24] Zhang H., Zhao S., Xin Z., Ye C., Li Z., Xia J. (2019). Ind. Eng. Chem. Res..

[cit25] Bouhelal S., Cagiao M. E., Benachour D., Calleja F. J. B. (2007). J. Appl. Polym. Sci..

[cit26] ISO 10993-13, Biological Evaluation of Medical Devices – Part 13: Identification and Quantification of Degradation Products from Polymeric Medical Devices

[cit27] Farr N., Pashneh-Tala S., Stehling N., Claeyssens F., Green N., Rodenburg C. (2019). Macromol. Rapid Commun..

[cit28] Sies H. (2017). Redox Biol..

[cit29] Pospisil J. and Klemchuk P. P., Oxidation Inhibition in Organic Materials, CRC Press, 1st edn, 1989, p. 384

[cit30] Celina M., George G. A. (1993). Polym. Degrad. Stab..

[cit31] Clavé A., Yahi H., Hammou J. C., Montanari S., Gounon P., Clavé H. (2010). International Urogynecology Journal.

[cit32] Cozad M. J., Grant D. A., Bachman S. L., Grant D. N., Ramshaw B. J. (2010). J. Biomed. Mater. Res., Part B.

[cit33] Mary C., Marois Y., King M. W., Laroche G., Douville Y., Marton L., Guidoin R. (1998). ASAIO J..

[cit34] Puchtler H., Meloan S. N. (1985). Histochemistry.

[cit35] Obtained 09/05/2021, https://omnexus.specialchem.com/polymer-properties/properties/water-absorption-24-hours

[cit36] Radl S., Larisegger S., Suzzi D., Khinast J. G. (2011). J. Pharm. Innovation.

[cit37] Vettegren V. I., Tshmel A. E. (1976). Eur. Polym. J..

[cit38] Rätzscha M., Arnold M., Borsigc E., Buckaa H., Reichelt N. (2002). Prog. Polym. Sci..

[cit39] Sokkar T., El-Farahaty K., Seisa E., Omar E., Agour M. (2014). Opt. Photonics J..

[cit40] Barone W. R., Amini R., Maiti S., Moalli P. A., Abramowitch S. D. (2015). J. Biomech..

[cit41] Liang R., Knight K., Abramowitch S., Moalli P.
A. (2016). Current Opinion in Obstetrics and Gynecology.

[cit42] Liang R., Abramowitch S., Knight K., Palcsey S., Nolfi A., Feola A., Stein S., Moalli P. A. (2013). BJOG.

[cit43] Chodák I. , Polymer Science and Technology Series. 2, 1992, Springer, Dordrecht, DOI: 10.1007/978-94-011-4421-6_18

[cit44] Kzhyshkowska J., Gudima A., Riabov V., Dollinger C., Lavalle P., Vrana N. E. (2015). J. Leukocyte Biol..

[cit45] Thompson M., Guelcher S., Bendavid R., Lakovlev V., Ostergard D. R. (2017). International Urogynecology Journal.

[cit46] Hwang J., Choi D., Han S., Choi J., Hong J. (2019). Sci. Total Environ..

[cit47] Wolf M. T., Dearth C. L., Ranallo C. A., LoPresti S. T., Carey L. E., Daly K. A., Brown B. N., Badylak S. F. (2014). Biomaterials.

[cit48] Verstraeten F., Gostl R., Sijbesma R. P. (2016). Chem. Commun..

