# Supplementary material for: A novel characterisation approach to reveal the mechano–chemical effects of oxidation and dynamic distension on polypropylene surgical mesh
Source: RSC Adv. 2021 Oct 27;11(55):34710–23. doi: 10.1039/d1ra05944k (PMC9042683; doi:10.1039/d1ra05944k)
Supplement: RA-011-D1RA05944K-s001 [file RA-011-D1RA05944K-s001.pdf]

Electronic Supplementary Material (ESI) for RSC Advances.
This journal is © The Royal Society of Chemistry 2021

A novel characterisation approach to reveal the mechano-chemical effects of oxidation and dynamic distension on polypropylene surgical mesh

Nicholas T.H. Farr[12], * Sabiniano Roman [1], Jan Schäfer[3], Antje Quade[3], Daniel Lester[4], Vanessa Hearnden, [1] Sheila MacNeil, [1] and Cornelia Rodenburg[1]

1 Department of Materials Science and Engineering, Sir Robert Hadfield Building, Mappin Street, University of Sheffield, UK.

2. Insigneo Institute for in *Silico* Medicine, The Pam Liversidge Building, Sir Robert Hadfield Building, Mappin Street, Sheffield, UK

3. Leibniz Institute for Plasma Science and Technology (INP e.V.), Felix-Hausdorff-Str. 2, 17489 Greifswald, Germany.

4. Polymer Characterisation Research Technology Platform, University of Warwick. Library Road, CV4 7AL. Coventry, UK

6. Supporting Information

6.1 Additional Methodology

High temp GPC:

All GPC data was recorded on an Agilent Technologies Infinity II 1260 HT GPC instrument equipped with differential refractive index (DRI), viscometry (VS) and dual angle light scatter (LS 90 + 15) detectors. The system was equipped with 2 x PLgel Olexis columns (300 x 7.5 mm) and a 10 µm guard column. The mobile phase was DCB with 250 PPM BHT (butylated hydroxytoluene) additive. Samples were run at 1 ml/min at 140’C. Polystyrene standards (Agilent EasyVials) were used to create a third order calibration between 6,570,000 – 580 $gmol^{-1}$. Analyte samples were dissolved at 140’C and hot filtered through a stainless steel frit with 10 μm pore size using an Agilent SP260VS to retain high temperature during filtration, before injection. Respectively, experimental molar mass ($Mn_{,SEC}$) and dispersity ($Đ$) values of synthesized polymers were determined by conventional calibration using Agilent GPC/SEC software.

X-ray photoelectron spectroscopy (XPS):

XPS measurements have been performed at different positions on the surface of both Gynemesh and Restorelle materials. For the determination of the chemical composition as a function the depth, XPS-depth profiles were recorded by means of gas cluster ion source sputtering. The analysis is performed by fitting the core level spectrum of C 1s after each sputtering cycle. Additionally, the elemental composition was determined from each measured point. The measurements were performed with the X-ray photoelectron spectrometer (XPS) Axis Supra, Kratos, Manchester, GB. The elemental distribution of the surface can be revealed by XPS imaging. Here, XPS images were acquired using the binding energy of C 1s signal. The images were taken with a resolution of 256x256 pixels over an area of 400x400 µm2. Small area spectroscopy can be performed from various areas of interest within an XPS image using a spot size of 55 µm. The measurement error is approximately ~5%. Two kinds of measurements were performed, survey scans and element scans (narrow scan). The spectra were recorded by means of monochromatic Al Kα excitation (1,486.6 eV) and magnetic immersion lens with charge neutralization (225 W X-ray radiation (15 kV, 15 mA), 40 eV pass energy.

The elemental composition was measured as a function of depth by alternating XPS measurements combined with a gas cluster ion source in cluster mode. During the sputter process the surface is exposed to a bombardment with a beam of Ar1000+ cluster at energies of 10 keV and timing which results in etching of the material from the sample surface. After this sputtering the composition is analysed before the next sputtering cycle starts. Consequently, a depth profile is obtained. The etched area was 1x1 mm2. The total etching time was 270 s. Data acquisition and processing were carried out using CASAXPS software, version 2.15 (Casa Software Ltd., UK).The highly resolved measured C 1s peak at 285.0 eV allows to quantify non-oxidized (C-C) and oxidized components of PP chains: hydroxyls (-COH, 286.5 eV), keto (C=O, 287.8 eV), and carboxyl acid (-COOH, 288.8 eV) groups.

6.2 Additional Results

The DRi chromatograms of the gynemesh samples are displayed in Figure S1 and S2 below (full chromatogram and zoomed to region of interest). The peaks at 10-12 and 12-15 minutes are attributed to the samples. In dichlorobenzene, polypropylene almost exclusively displays

negative polarity in differential refractive index detectors (very low molecular weights the exception). Because of this the 12-15 minutes peak is likely due to the dissolved polypropylene from the Gynemesh. The earlier peak at 10-12 minutes is positive and elutes before the PP peak, suggesting it is larger. The positive polarity of this peak could suggest that it is either of a different chemical composition to PP, or that it is insoluble particles that have traversed the GPC column, which often lead to positive peaks in DRi detectors.

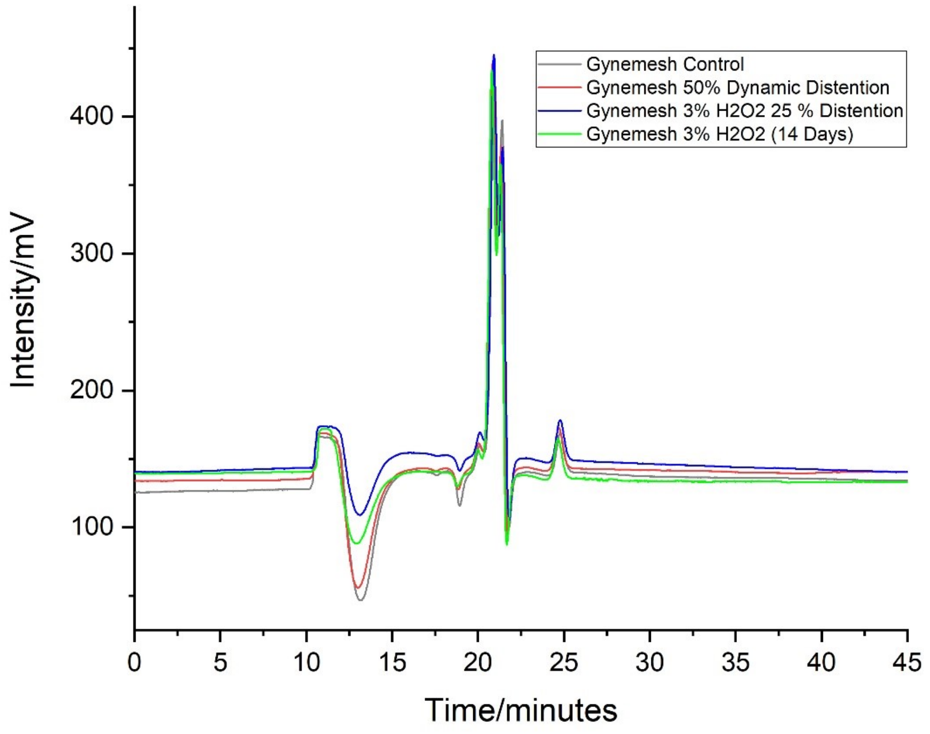

Figure S1: Full DRi chromatograms of Gynemesh control, Gynemesh after 50% distention, Gynemesh in 3% H202 with 25% distention and Gynemesh exposed to H202 alone.

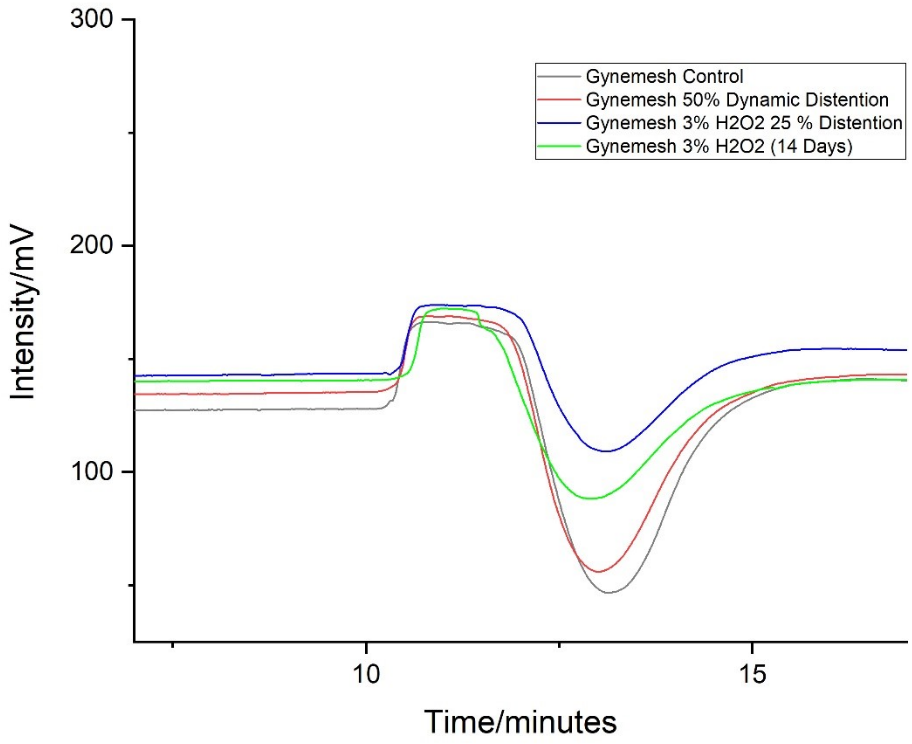

Figure S2: DRi chromatograms (0 – 20 min region of interest) of Gynemesh control, Gynemesh after 50% distention, Gynemesh in 3% H202 with 25% distention and Gynemesh exposed to H202 alone.

The molecular weight distributions are presented in Figure S3, where the suspected insoluble material can be clearly observed at very high molecular weights. Note that due to the DRi detector being the concentration detector, and its reliance on dn/dc, that insoluble materials will not lead to valid molecular weight data, however, qualitatively we can suggest that this suspected particulate is present across the 4 samples.

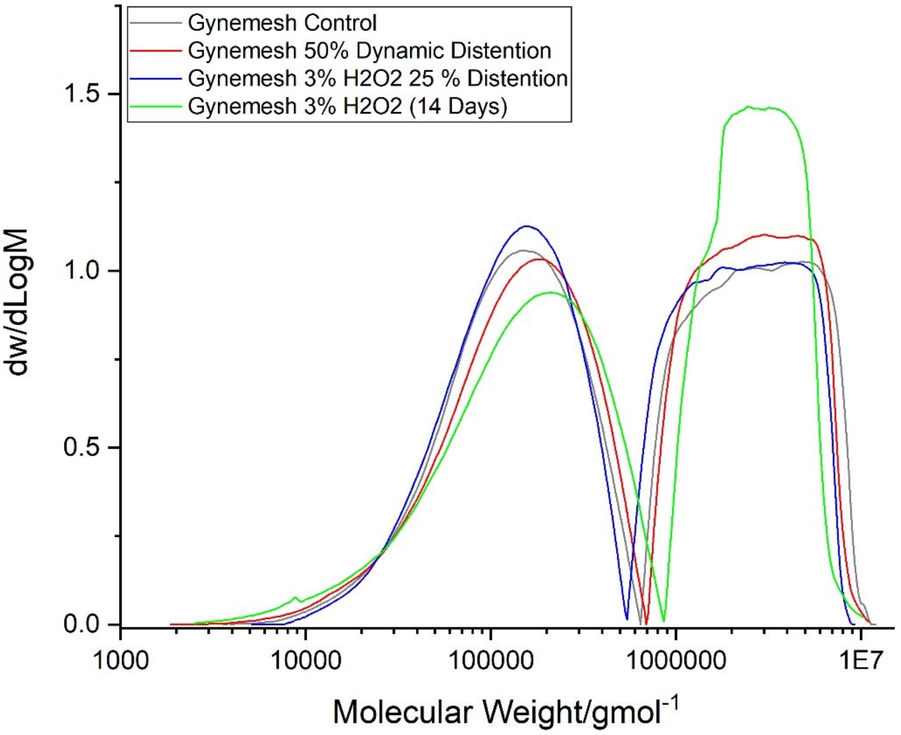

Figure S3: Molecular weight distributions of Gynemesh control, Gynemesh after 50% distention, Gynemesh in 3% H202 with 25% distention and Gynemesh exposed to H202 alone.

The weight average molecular weights (M_w) and dispersities are presented in the figure S4 below, where it can be seen that the control and 25% distention display the lowest molecular weights, however, the highest molecular weight sample, 14 days, was only slightly higher in weight average molecular weight than the others. This sample also displayed the highest dispersity. This slightly lower M_w and PDi may suggest that samples which underwent dynamic distention degraded more than samples only treated with H202.

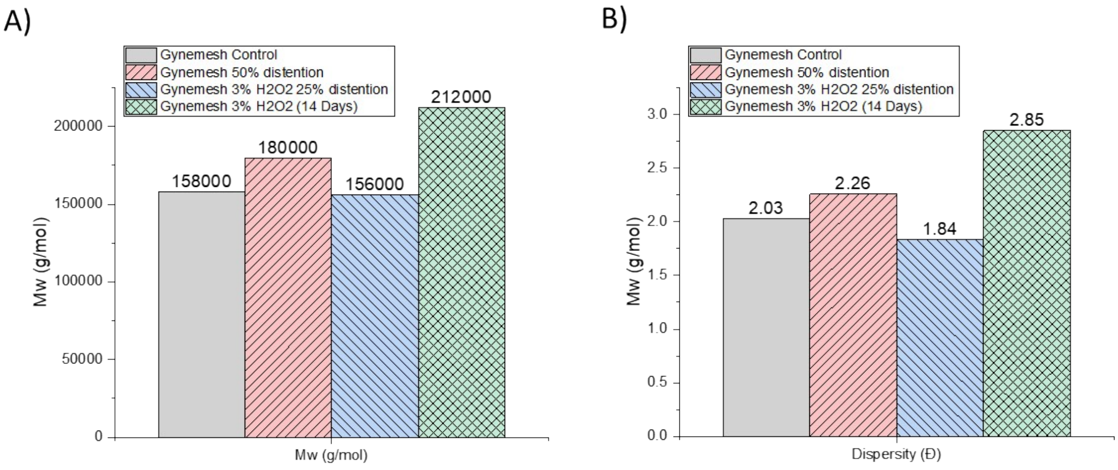

Figure S4: A) Weight average molecular weights (Mw) and B) Dispersity (Đ) of Gynemesh control, Gynemesh after 50% distention, Gynemesh in 3% H202 with 25% distention and Gynemesh exposed to H202 alone.
